# Gene Therapy in Thalassemia and Hemoglobinopathies

**DOI:** 10.4084/MJHID.2009.008

**Published:** 2009-11-13

**Authors:** Laura Breda, Roberto Gambari, Stefano Rivella

**Affiliations:** 1 Weill College Medical Center, Department of Pediatrics, Division of Hematology-Oncology, NY, USA; 2 Department of Biochemistry and Molecular Biology, Section of Molecular Biology, University of Ferrara, Italy

## Abstract

Sickle cell disease (SCD) and ß-thalassemia represent the most common hemoglobinopathies caused, respectively, by the alteration of structural features or deficient production of the ß-chain of the Hb molecule. Other hemoglobinopathies are characterized by different mutations in the α- or ß-globin genes and are associated with anemia and might require periodic or chronic blood transfusions. Therefore, ß-thalassemia, SCD and other hemoglobinopathies are excellent candidates for genetic approaches since they are monogenic disorders and, potentially, could be cured by introducing or correcting a single gene into the hematopoietic compartment or a single stem cell. Initial attempts at gene transfer of these hemoglobinopathies have proved unsuccessful due to limitations of available gene transfer vectors. With the advent of lentiviral vectors many of the initial limitations have been overcame. New approaches have also focused on targeting the specific mutation in the ß-globin genes, correcting the DNA sequence or manipulating the fate of RNA translation and splicing to restore ß-globin chain synthesis. These techniques have the potential to correct the defect into hematopoietic stem cells or be utilized to modify stem cells generated from patients affected by these disorders. This review discusses gene therapy strategies for the hemoglobinopathies, including the use of lentiviral vectors, generation of induced pluripotent stem cells (iPS) cells, gene targeting, splice-switching and stop codon readthrough.

## ß-thalassemia, sickle cell anemia and other hemoglobinopathies:

The thalassemias are a group of disorders due to a large number of heterogeneous mutations causing abnormal globin gene expression resulting in the total absence or quantitative reduction of globin chain synthesis^1^. Mutations in the α- or ß-globin gene lead to α- and ß-thalassemia, respectively^1^. α-Thalassemia is usually due to deletions within the α-globin gene cluster, leading to loss of function of one or both α-globin genes in each locus^2^. However, non-deletion mutations have been described, although they are much less frequent^1^. Depending on the number of genes that are unable to synthesize the α-globin protein, different clinical manifestations can be observed. If one or two α-globin genes are mutated (in *cis* or *trans*), normally no or minimal hematological effects are seen, and individuals are normally silent thalassemia carriers or show α-thalassemia trait^1^. If three out of four genes are mutated, the condition is called hemoglobin H (HbH) disease, resulting in a hemolytic anemia that can worsen with febrile illness or exposure to certain drugs, chemicals, or infectious agents. Hemoglobin H disease is characterized by moderate to severe anemia, hepatosplenomegaly, and jaundice. Transfusion may occasionally be required and, if provided frequently, can lead to iron overload. If all four α-globin genes are deleted, the resulting condition is called α-thalassemia major, which is so severe that death occurs in utero. Children rescued through intrauterine transfusions remain dependent on red blood cell transfusions for survival^3^.

The thalassemias are characterized by their clinical severity and genetic mutations. Patients with Cooley’s anemia, also known as ß-thalassemia major, which is the most severe form of this disease, require many blood transfusions per year and is characterized by ineffective erythropoiesis and extra medullary hematopoiesis (EMH)^1^. If untreated, ß-thalassemia major is fatal in the first few years of life^1^. In ß-thalassemia intermedia, where a greater number of ß-globin chains are synthesized, the clinical picture is milder, and the patients require only infrequent or no transfusions^4^,^1^. In both thalassemias, with time the spleen is enlarged, the hemoglobin level decreases, and progressive iron overload occurs from increased GI iron absorption in addition to transfusions^1^. The vast majority of ß-thalassemias are caused by point mutations within the gene or its immediate flanking sequences and are classified according to the mechanism by which they affect gene regulation: transcription, RNA processing and mRNA translation^1^. These mutations are also classified as ß0 and ß+ according to the quantity of ß-globin chains synthesized. Mutations that lead to alternative splicing are associated with reduced synthesis of normal ß-globin mRNA and protein and are defined ß+. In contrast, mutations that completely impair ß-globin synthesis (for instance premature termination codons or PTCs) are defined ß0. Depending on the association of these different mutations, patients are classified into three principal groups with none, very low or low ß-globin production (ß0/0, 0/+, +/+ respectively). The levels of fetal hemoglobin (HbF) account for a large part of the clinical heterogeneity observed in patients with ß-thalassemia. Variation in HbF expression among individuals is an inheritable disease modifier and high HbF (composed from 2 α- and 2 γ-chains) levels generally correlate with reduced morbidity and mortality in this disorder, since the γ-globin chains combine with the excess α-chains.

A single mutation leads to SCD, causing an adenine (A) to thymidine (T) substitution in codon 6 (GAG-GTG), which leads to insertion of valine in place of glutamic acid in the ß-globin chain. The resulting Hb (HbS) has the unique property of polymerizing when deoxygenated^1^. When the polymer becomes abundant, the red cells “sickle”, stiff rods form that stretch and distort the red cells. These distorted cells can obstruct blood flow through the small vessels, and the restricted oxygen delivery to the tissues damages cells, injures organs, and produces pain. Similarly to SCD, other hemoglobinopathies can be triggered by the substitution of one amino acid (HbE^5^,^6^,^2^), deletion of a portion of the amino acid sequence (Hb Gun Hill^7^), abnormal hybridization between two chains (Hb Lepore^8^,^9^), or abnormal elongation of the globin chain (Hb Constant Spring^10^). These abnormal Hbs can have a variety of pathophysiologically significant effects, including ineffective erythropoiesis and anemia^1^.

SCD and the thalassemias are quite common among Asian, African, African-American and Mediterranean populations^1^. It has been estimated that approximately 7% of the world population are carriers of such disorders, and that 300,000–400,000 children with severe forms of these diseases are born each year^117^.

## Hematopoietic stem cell transplantation:

Current disease management of ß-thalassemia consists of prenatal diagnosis, transfusion therapy, or allogeneic BMT^11^–^13^. Only the latter is potentially curative^14^. The first successful BMT of ß-thalassemia was reported in 1982^15^. Consequently, several centers have utilized this approach as definitive therapy^16^–^18^. The most extensive experience in treating ß-thalassemia patients with BMT is that of Lucarelli and coworkers in Italy^18^. Established protocols can lead to a high success of thalassemia-free survival, although the transplant-related mortality is still significant and the chronic graft-versus-host disease is still a potential long-term complication of allogeneic HSCs transplantation^17^,^19^. In addition, availability of allogeneic bone marrow is limited by finding an identical human leucocyte antigen (HLA) matched bone marrow donor. However, development of new techniques to improve the management of graft-versus-host disease, to perform BMT from unrelated donors and cord blood stem cells may expand the pool of potential donors in the near future^20^.

In addition, patients with severe ß-thalassemia and SCD might benefit from new genetic and cellular approaches. From this prospective, ß-thalassemia and SCD are excellent candidate diseases for genetically based therapies in autologous hematopoietic stem cells (HSCs)^21^–^23^. Alternatively, somatic cells reprogrammed to induced pluripotent stem cells might also provide a possible new approach to treat ß-thalassemia^24^,^25^.

## Gene transfer using oncoretroviral vectors:

Gene addition mediated by retroviral vectors is an attractive approach for monogenic disorder, However, when applied to hemoglobinopathies, this strategy raises major challenges in terms of controlling transgene expression, which should be erythroid-specific, elevated, position independent and sustained over time. In fact, many studies were performed before positive preclinical data were generated. The first attempts were done using oncoviruses. These viruses belong to the large family of Retroviridae and are characterized by a genome that encodes the genes gag-pol and env^26^. Onco-retroviral vectors, such as those derived from Moloney murine leukemia virus, efficiently transfer therapeutic genes into murine hematopoietic stem cells (HSC) without transferring any viral gene^27^. Recombinant oncoretroviruses were the first viral vectors used to transfer the human ß-globin gene in mouse HSCs^28^,^29^. These experiments resulted in tissue-specific but low and variable (position-dependent) human ß-globin expression in bone marrow chimeras, usually varying between 0 and 2% of endogenous mouse ß-globin mRNA levels^29^,^30^–^33^. Studies aimed at increasing expression levels of transferred ß-globin genes have focused on including locus control region (LCR) elements of the human ß-globin gene locus into oncoretroviral vectors. The LCR contains cis-acting DNase I hypersensitivity sites (HS) that are critical for high-level, long-term, position-independent, and erythroid-specific expression^34^,^35^. These HS elements contain several DNA-binding motifs for transcriptional and chromatin remodeling factors that facilitates chromatin opening. Also, thes genomic regions allow for binding of other regulatory elements required for high-level expression of the ß-globin gene^36^. Incorporation of the core elements of HS2, HS3, and HS4 of the human ß-globin LCR significantly increased expression levels in murine erythroleukemia (MEL) cells but failed to abolish positional variability of expression^37^,^35^. Additional efforts aimed to include larger elements resulted in the inability of the vector to incorporate large quantities of genetic material, as shown by the rearrangements of the transferred sequences^38^–^41^. Since these rearrangements frequently occur because of activation of splicing sites of the LCR sequence contained in the retroviral RNA, additional attempts were done to eliminate these sites. However, even these new vectors failed to include HS elements sufficient large to considerably increase expression of the ß-globin gene^37^,^35^.

Additional erythroid-specific transcriptional elements were investigated within oncoretroviral vectors, including the HS40 regulatory region from the human α-locus^42^–^44^ and alternative promoters. The promoter of ankyrin, a red cell membrane protein, has shown some promise in transgenic mice and in transduced MEL cells^45^. In mice, the ankyrin promoter has been used to drive expression of the human γ-globin gene resulting, at double copy, in an average expression of 8% of that of the endogenous α-globin genes^46^. To overcome transcriptional silencing of the γ-globin promoter in hematopoietic chimeras, mutant γ-globin promoters from patients with hereditary persistence of fetal hemoglobin (HPFH) were also investigated^118^,^47^. The Greek mutation at position 117 thus appeared to substantially increase γ-globin expression in MEL cells^47^. However, even these vectors failed to increase the level of the ß-globin gene to therapeutic levels.

Although oncoretrovirus vectors integrate into the genome, many integrants undergo transcriptional silencing, posing an additional challenge to the success of gene therapy using these vectors. Kalberer and co-workers attempted to avoid gene silencing by preselecting ex vivo retrovirally transduced hematopoietic stem cells on the basis of expression of the green fluorescent protein (GFP). In this vector the GFP gene was driven by the phosphoglycerate kinase promoter, while the human ß-globin gene by its own promoter and small elements from the LCR^48^. Using this approach, *in vivo* hematopoietic stem cell gene silencing and age-dependent extinction of expression were avoided, although suboptimal expression levels and heterocellular position effects persisted.

Another major limitation is that oncoretroviral vectors need to infect cells before and close to their division, otherwise the viral RNA cannot migrate into the nucleus due to the presence of a nuclear membrane^49^. Since most hematopoietic stem cells are in a quiescent state, they must be induced with cytokines to divide in order to achieve higher transduction efficiencies and overall expression levels. Stimulation of quiescent hematopoietic stem cells, however, impairs or halts their long-term repopulating capacities^49^.

## Gene Transfer Using Lentiviral Vectors:

With the extensive research on human immunodeficiency virus-1, it has been realized that lentivirus, engineered to be devoid of any pathogenic elements, can become efficient gene transfer vectors. Lentiviruses are characterized by a complex genome that encodes a number of accessory proteins besides the canonical retroviral genes gag-pol and env. They share all the common characteristic of retroviral replication including receptor-mediated entry, capsid uncoating, reverse transcription of the viral RNA, and integration into the host cell genome^26^. In addition, they are able to transduce non-replicating cells, which confers to these viruses a special value for the development of clinically functional gene vectors. Moreover, compared to oncoretroviral vectors, the stabilization of the proviral mRNA genome by the interaction of the accessory protein Rev with its cognate motif Rev-responsive element (RRE), increases their range of application, since larger genomic elements can be introduced in their genome with limited or no sequence rearrangement^50^. Therefore, lentiviral vectors are thus likely to be selected as vectors of choice for the stable delivery of regulated transgenes in stem cell–based gene therapy. The use of lentiviral vectors has allowed the introduction of large genomic elements from the ß-globin locus, different promoters, enhancers, and chromatin structure determinants that led to lineage-specific and elevated of ß-, γ- and α-globin expression *in vivo*. This resulted, in the amelioration or correction of anemia and secondary organ damage in several murine models of hemoglobinopathies, making the recombinant lentiviruses the most effective vector system to date for gene therapy of these disorders.

α-Thalassemia could potentially be a target for fetal gene therapy since fetuses with this disorder usually die between the third trimester of pregnancy and soon after birth. The potential use of lentiviral vectors to treat α-thalassemia was investigated a vector containing the HS2, 3, and 4 of the LCR from the human ß-globin locus, and the human α-globin gene promoter directing the human α-globin gene. Using this vector, Han and colleagues performed gene delivery *in utero* during midgestation targeting embryos affected by a lethal form of α-thalassemia. They showed that in newborn mice, the human α-globin gene expression was detected in the liver, spleen, and peripheral blood^51^. The human α-globin gene expression was at the peak at 3–4 months, when it reached 20% in some recipients. However, the expression declined at 7 months. Colony-forming assays in these mice showed low levels of transduction and lack of human α-globin transcript. Thus, lentiviral vectors can be an effective vehicle for delivering the human α-globin gene into erythroid cells *in utero*, but, in the mouse model, delivery at late midgestation could not transduce hematopoietic stem cells adequately to sustain gene expression.

Treatment of ß-thalassemia, SCD and other disorders through lentiviral mediated gene transfer is studied in murine and primate models^52^–^60^. The original studies in mice showed that lentiviral mediated human ß-globin gene transfer can rescue mice affected by ß-thalassemia intermedia and ß-thalassemia major^61^,^62^,^59^. The mouse ß-globin cluster has two adult ß-globin genes, ß^minor^- and ß^major^-globin. Thalassemic mice were generated with deletion of both the ß^minor^- and ß^major^-globin on one allele, designated *th3*/+ mice (63; 64). Also adult *th3*/+ mice have a degree of disease severity (hepatosplenomegaly, anemia, aberrant erythrocyte morphology) comparable to that of patients affected by ß-TI. May and colleagues tested two lentiviral vectors termed RNS1 (carrying minimal core LCR elements) and TNS9 (with large LCR fragments encompassing HS2, HS3 and HS4; approximately 3.2 kb in size) on *th3*/+ mice. Compared to RNS1, mice recipient of the larger TNS9 vector maintained higher human ß-globin transcript levels over time showing amelioration of red cell pathology (anisocytosis and poikilocytosis) and significantly increased hemoglobin levels (from 8–9 g/dL to 11–13 g/dL). The massive splenomegaly found in chimeras engrafted with control *th3*/+ bone marrow was not observed in TNS9-treated animals^61^. This correction was sustained in secondary mice^62^.

Mice completely lacking adult ß-globin genes (*th3*/*th3*) die late in gestation, limiting their utilization as a model for Cooley’s anemia^64^. For this reason, adult animals affected by Cooley’s anemia were generated by transplantation of hematopoietic fetal liver cells harvested from *th3*/*th3* embryos at E14.5 into lethally irradiated syngeneic adult recipients^59^. Hematological analyses of engrafted mice performed 6 to 8 weeks post-transplant revealed severe anemia due not to pancytopenia but rather to low red blood cell and reticulocyte counts together with massive splenomegaly and extensive EMH^62^,^59^. These animals could be rescued using TNS9 or by blood transfusions, supporting the notion that their phenotype is due specifically to erythroid impairment^65^,^59^.

Pawliuk and colleagues investigated the efficacy of a lentiviral vector harboring the ß-globin promoter, LCR elements and a mutated human ß-globin gene with enhanced anti-sickling activity (ß87) in two different transgenic mouse models for SCD: SAD and BERK^66^,^67^. Mice transplanted with BERK and SAD bone marrow cells transduced with this modified ß-globin gene exhibited corrected reticulocyte counts and amelioration of Hemoglobin concentration, anisocytosis, and poikilocytosis. Moreover, the proportion of irreversibly sickled cells, SCD-associated splenomegaly, and characteristic urine concentration defect in SAD and BERK mice were vastly improved or corrected by ß87. Using a similar vector, Levasseur and colleagues obtained equivalent results. They transduced Sca1+c-Kit+Lin− cells rather than unselected bone marrow cells and achieved durable therapeutic results (5–7 months) following transplantation of 100 cells in lethally irradiated C57BL/6 mice^113^,^114^.

Samakoglu and coworkers applied the principle of RNA interference (RNAi) to down-regulate the ß-globin mRNA in CD34(+) cells from patients affected by SCD^116^. They utilized a lentiviral vector harboring a promoterless small-hairpin RNA (shRNA) within the intron of a recombinant γ-globin gene. Expression of both γ-globin and the lariat-embedded small interfering RNA (siRNA) was induced upon erythroid differentiation, specifically downregulating the targeted gene in tissue and differentiation stage-specific fashion. The position of the shRNA within the intron was critical to concurrently achieve high transgene expression, effective siRNA generation and minimal interferon induction.

Miccio and colleagues also utilized an erythroid-specific lentiviral vector driving the expression of the human ß-globin gene from a minimal promoter/enhancer element containing two hypersensitive sites from the ß-globin locus control region in mouse models of ß-thalassemia (68). They showed that genetically corrected erythroblasts underwent *in vivo* selection. The selected erythroblast that derived from progenitors harboring proviral integrations in genome sites and were more favorable to high levels of vector expression. These data suggested that a regimen of partially myeloablative transplantation might be sufficient to achieve a chimerism that would therapeutic in ß-thalassemic patients.

While correction of murine models of ß-thalassemia has been achieved through lentiviral-mediated high levels of globin gene transfer into mouse HSCs, transduction of human HSCs is less robust and may be inadequate to achieve therapeutic levels of genetically modified erythroid cells. Zhao and coworkers therefore developed a double gene lentiviral vector encoding both human γ-globin under the transcriptional control of erythroid regulatory elements and methylguanine methyltransferase (MGMT), driven by a constitutive cellular promoter^60^. MGMT is an alkyltransferase that normally functions to repair cellular DNA damage at the O^6^ position of guanine^69^,^70^. The cytotoxic effects of alkylating agents, such as temozolomide and 1,3-bis-chloroethyl-1-nitrosourea (BCNU), can be prevented if there is adequate expression of MGMT, which removes the O^6^ adduct from the modified DNA. Variant MGMT proteins with specific amino acid changes retain significant activity while possessing the useful property of resistance to inactivation by O^6^-benzylguanine (BG)^71^. BG can be used to inactivate endogenous MGMT to enhance the specificity of alkylator-mediated cell death to cells not expressing the variant form. Therefore, expression of these variant forms of MGMT provides cellular resistance to alkylator drugs, which can be administered to kill residual untransduced HSCs, whereas transduced cells are protected. To test this hypothesis, mice transplanted with ß-thalassemic HSCs cells transduced with a lentiviral γ-globin/MGMT vector were treated with BCNU^60^. This led to significant increas in the number of γ-globin–expressing red cells, the amount of fetal hemoglobin and resolution of anemia. One important advantage of using the γ-globin gene, normally expressed exclusively during fetal life, is that high level γ-globin expression would be therapeutic not only for ß-thalassemia, but also SCD. Interestingly, selection of transduced HSCs was also obtained when cells were drug-treated before transplantation. These data suggest that coexpression of MGMT allowed autologous, γ-globin vector-transduced ß-thalassemic HSCs to be enriched to therapeutic levels through either pre or post-transplantation selection.

Imren and colleagues engrafted immunodeficient mice with human cord blood cells infected with a lentiviral vector encoding an anti-sickling ß-globin transgene^35^,^72^. After 6-months, half of the human erythroid and myeloid progenitors regenerated in the mice containing the transgene, and erythroid cells derived *in vitro* from these cells produced high levels of the ß-globin protein. In addition, these authors investigated the integrated proviral copies showing that 86% of the proviral inserts had occurred within genes, including several genes implicated in human leukemia. These findings indicate effective transduction of very primitive human cord blood cells achieving robust and erythroid-specific production of therapeutically relevant levels of ß-globin protein. The frequency of proviral integration within genes observed in this study and the data from Miccio and coworkers that indicate that selected erythroblasts were derived from progenitors harboring proviral integrations more favorable to high levels of vector expression, indicate that regulated hematopoiesis might require additional safety modifications to prevent potential genotoxic effects^35^,^72^,^68^. This risk is inherent to the integration of foreign genetic material and the risk of insertional oncogenesis has been established both in mice and humans^73^–^78^.

**Figure 1.**
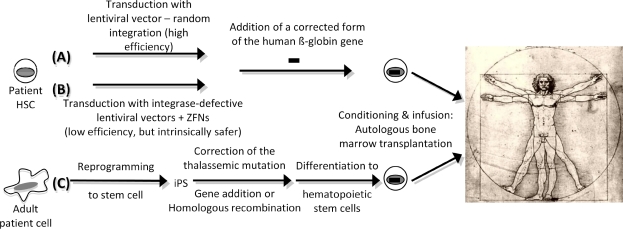
Schematic representation of the gene therapy approach mediated, respectively, by (A) gene transfer into hematopoietic stem cells (HSC) using integration competent lentiviral vector (B) gene transfer into HSC by integrase defective lentiviral vectors. ZFN: zinc finger protein. (C) Stem cell therapy by reprogramming of adult cells to stem cells. iPS: Induced Pluripotent Stem Cell.

In light of these results, genetic elements with enhancer-blocking properties, such as insulators, could increase the safety of the clinical trails. These elements have been investigated to shelter the vector from the repressive influence of flanking chromatin by blocking interactions between regulatory elements within the vector and chromosomal elements at the site of integration^79^–^81^. This property of insulators can also be harnessed to diminish the risk that the vector will activate a neighboring oncogene^82^,^83^. The initial studies indicated that inclusion of the cHS4 insulator element into the 3′ LTR of recombinant murine leukemia virus increases the probability that randomly integrated proviruses will express the transgene^46^,^84^–^86^. Puthenveetil and coworkers tested a lentiviral vector carrying the human ß-globin expression cassette flanked by a chromatin insulator in transfusion-dependent human ß-thalassemia major cells^87^. Using this vector, they demonstrated normal expression of human ß-globin in erythroid cells produced *in vitro*. They also observed restoration of effective erythropoiesis and reversal of the abnormally elevated apoptosis that characterizes ß-thalassemia. The gene-corrected human ß-thalassemia progenitor cells were also transplanted into immune-deficient mice, where they underwent normal erythroid differentiation, expressed normal levels of human ß-globin, and displayed normal effective erythropoiesis 3 to 4 months after xenotransplantation. Based on all these preclinical studies on mouse models of ß-thalassemia and SCD, clinical trials have been proposed or are underway^53^. Figure 1A depicts this approach.

Alternatively, the homologous recom-bination pathway can be harnessed to avoid random integration. Zinc-finger nucleases (ZFNs) can been used to enhance the frequency of gene correction^88^,^89^. However, achieving the full potential of ZFNs for genome engineering in human cells requires their efficient delivery to the relevant cell types. Lombardo and colleagues exploited the infectivity of integrase-defective lentiviral vectors (IDLV) to express ZFNs and provide the template DNA for gene correction in different cell types^90^. IDLV-mediated delivery supported high rates (13–39%) of editing at the IL-2 receptor common γ-chain gene (*IL2RG*) across different cell types as well as human embryonic stem cells (5%), allowing selection-free isolation of clonogenic cells with the desired genetic modification. Therefore, this technique opens new and exciting possibilities. By modifying the ZFN binding specificity and selecting an appropriate donor sequence, one could target the IDLV-ZFN system to any individual site in the human genome avoiding random integration (Figure 1B) and, potentially, genome toxicity^88^–^91^.

However, there are current obstacles to successfully apply this therapeutic approach to humans. Some of them include the need for improved efficiency of gene delivery, insertion of the gene into non-oncogenic sites and the potential negative or positive contributions of the ß-thalassemic genotype and potential modifiers to the effectiveness of the gene transfer^1^. Original studies in animal models utilized mice with deletions of the ß-globin genes. These mutations do not reflect the phenotypic variability observed in ß-thalassemic patients. Thus, there is a gap in knowledge between our understanding of the primary mutation, the corresponding phenotype, and the approach to cure an individual patient based on his/her genotype (*i.e.* understanding of the disease and its treatment by genetic modalities). To date this variability has not been addressed and no studies have focused on the efficacy of gene therapy in relation to the different genotypes of the patients. Although gene therapy is an area of active clinical investigation, the aforementioned obstacles limit its use in the management of thalassemia. Nonetheless, as we showed in our review the successful transfer of globin genes into hematopoietic cells of humans has been demonstrated and is encouraging.

## Gene Correction and Ips Cells:

Triplex-forming oligonucleotides and triplex-forming peptide nucleic acids (PNAs) have been shown to stimulate recombination in mammalian cells via site-specific binding and creation of altered helical structures that provoke DNA repair^92^,^93^. Cotransfection of PNAs and recombinatory donor DNA fragments, Chin and co-workers demonstrated that these complexes can promote single base-pair modification at the start of the second intron of the beta-globin gene, the site of a common thalassemia-associated mutation^94^. This single base pair change was detected by the restoration of proper splicing of transcripts produced from a green fluorescent protein-beta-globin fusion gene. The ability of these PNAs to induce recombination was dependent on dose, sequence, cell-cycle stage, and the presence of a homologous donor DNA molecule. They also showed that these PNAs were effective in stimulating the modification of the endogenous beta-globin locus in human cells, including primary hematopoietic progenitor cells. Enhanced recombination, however, did not exhibit frequencies superior to 0.4%^94^. However, this technology could be a powerful tool in combination with the generation of stem cells. In particular, introduction of the genes Oct3/4, Sox2 with either Klf4 and c-Myc or Nanog and Lin28 genes can induced pluripotent stem (iPS) cells^95^,^115^,^24^,^96^. Ye and coworkers shown that iPS cells can be generated from cells derived from skin fibroblasts, amniotic fluid or chorionic villus sampling of patients with ß-thalassemia^97^. Subsequently, the iPS cells were differentiated into hematopoietic cells that synthesized hemoglobin. Therefore, in the future the mutation in the ß-globin gene of these iPS cells could be corrected by gene targeting and the cells differentiated into HSCs to be returned to the patient^94^. Figure 1C depicts this approach. In fact, mice affected by SCD were cured using this strategy^98^. However, there are some obstacles that need to be overcome before iPS treatment of ß-thalassemia will be utilized. One of the most pressing problems is elimination of the transcription factors when they are no longer needed. Second, it is necessary to reestablish the correct reprogramming so that the iPS cells do not develop into tumors.

## Splice-Switching and Stop Codon Readthrough:

Defective ß-globin gene expression and ß-globin deficiency can be attributed to almost 200 thalassemic mutations. However, only 10 mutations are responsible for the majority of cases worldwide and some of the most frequent cause aberrant splicing of intron 1 (IVS1-110, IVS1-6, IVS1-5) or intron 2 (IVS2-654, IVS2-745)^99^,^112^. These mutations lead to incorrectly spliced mRNAs, even though the correct splice sites remain undamaged and potentially functional. Use of small nuclear RNA (snRNA) and splice-switching oligonucleotides represents a promising approach since these molecules can restore the corrected splicing re-establishing the synthesis of the normal protein^94^,^100^–^108^. Therefore blocking the aberrant splice sites with antisense oligonucleotides forces the splicing machinery to reselect the existing correct splice sites. Expression of antisense sequences targeted to the aberrant splice sites in thalassemic pre-mRNA has been successful, restoring the correct splicing pattern and ultimately restoring hemoglobin synthesis^102^,^93^. This was demonstrated in HSCs and erythroid progenitor cells from a patient with IVS2-745/IVS2-1 thalassemia. After transduction of the patient cells with a lentiviral vector that express a snRNA targeting the mutant RNA, the levels of correctly spliced ß-globin mRNA and adult hemoglobin were approximately 25-fold over baseline^108^. Similarly, the correct splicing pattern was restored in a mouse model of IVS2-654 thalassemia. This was achieved by delivery *in vivo* of a splice-switching oligonucleotide, a morpholino oligomer conjugated with an arginine-rich peptide. Repaired ß-globin mRNA restored significant amounts of hemoglobin in the peripheral blood of the IVS2-654 mouse, improving the number and quality of erythroid cells^107^.

Another approach showing a great potential for the treatment of genetic disorders characterized by to premature termination codons (PTCs) is the use of drugs to induce stop codon readthrough. These modified RNA would protected against non-sense mediated mRNA decay (NMD) and allow production of a protein^109^. Aminoglycoside antibiotics can decrease the accuracy in the codon-anticodon base pairing, inducing a ribosomal read through of premature termination codon. These findings have led to the development of a pharmacologic approach to treat thalassemic patients carrying the ß0–39 mutation, which introduces a PTC in codon 39 of the ß-globin gene and is one of the most frequent thalassemic mutations in the Mediterranean littoral^1^. Aminoglycosides and analogous molecules were tested in their ability to restore ß-globin protein synthesis on human erythroid cells (K562) carrying a lentiviral construct containing the ß0–39 globin-gene^110^. Treatment of these cells with geneticin (G418) and other aminoglycosides restored the production of ß-globin^110^. Moreover, after FACS and high performance liquid chromatography (HPLC) analyses, G418 was also demonstrated to partially correct the biological function of the ß0–39 globin mRNA in erythroid precursor cells from ß0–39 homozygous thalassemia patients^111^. This study strongly suggests that ribosomal read-through should be considered a novel approach for treatment of ß0 thalassemia caused by premature stop codon mutations and NMD.

## References

[1] Adamkiewicz TV Szabolcs P Haight A Baker KS Staba S Kedar A Chiang KY Krishnamurti L Boyer MW Kurtzberg J Wagner JE Wingard JR Yeager AM 2007 Unrelated cord blood transplantation in children with sickle cell disease: review of four-center experience Pediatr Transplant 11 6 641 644 17663687 10.1111/j.1399-3046.2007.00725.x

[2] Arumugam PI Scholes J Perelman N Xia P Yee JK Malik P 2007 Improved human beta-globin expression from self-inactivating lentiviral vectors carrying the chicken hypersensitive site-4 (cHS4) insulator element Mol Ther 15 10 1863 1871 17622240 10.1038/sj.mt.6300259

[3] Bank A Dorazio R Leboulch P 2005 A phase I/II clinical trial of beta-globin gene therapy for beta-thalassemia Ann N Y Acad Sci 1054 308 316 16339679 10.1196/annals.1345.007

[4] Bell AC West AG Felsenfeld G 1999 The protein CTCF is required for the enhancer blocking activity of vertebrate insulators Cell 98 3 387 396 10458613 10.1016/s0092-8674(00)81967-4

[5] Bell AC West AG Felsenfeld G 2001 Insulators and boundaries: versatile regulatory elements in the eukaryotic Science 291 5503 447 450 11228144 10.1126/science.291.5503.447

[6] Bender MA Gelinas RE Miller AD 1989 A majority of mice show long-term expression of a human beta-globin gene after retrovirus transfer into hematopoietic stem cells Molecular and cellular biology 9 4 1426 1434 2657395 10.1128/mcb.9.4.1426-1434.1989 PMC362559

[7] Beutel G Meyer J Ma L Yin S Eder M von Neuhoff N Wilkens L Wei J Hertenstein B Heil G Schlegelberger B Ganser A Li Z Baum C 2005 Expression of the p75 neurotrophin receptor in acute leukaemia Br J Haematol 131 1 67 70 16173964 10.1111/j.1365-2141.2005.05717.x

[8] Bibikova M Carroll D Segal DJ Trautman JK Smith J Kim YG Chandrasegaran S 2001 Stimulation of homologous recombination through targeted cleavage by chimeric nucleases Molecular and cellular biology 21 1 289 297 11113203 10.1128/MCB.21.1.289-297.2001 PMC88802

[9] Bodine DM Karlsson S Nienhuis AW 1989 Combination of interleukins 3 and 6 preserves stem cell function in culture and enhances retrovirus-mediated gene transfer into hematopoietic stem cells Proceedings of the National Academy of Sciences of the United States of America 86 22 8897 8901 2813429 10.1073/pnas.86.22.8897 PMC298397

[10] Boulad F Giardina P Gillio A Kernan N Small T Brochstein J Van Syckle K George D Szabolcs P O’Reilly RJ 1998 Bone marrow transplantation for homozygous beta-thalassemia. The Memorial Sloan-Kettering Cancer Center experience Ann N Y Acad Sci 850 498 502 9668595 10.1111/j.1749-6632.1998.tb10532.x

[11] Burgess-Beusse B Farrell C Gaszner M Litt M Mutskov V Recillas-Targa F Simpson M West A Felsenfeld G 2002 The insulation of genes from external enhancers and silencing chromatin Proceedings of the National Academy of Sciences of the United States of America 1 1 10.1073/pnas.162342499 PMC139905 12154228

[12] Bush S Mandel FS Giardina PJ 1998 Future orientation and life expectations of adolescents and young adults with thalassemia major Ann N Y Acad Sci 850 361 369 9668559 10.1111/j.1749-6632.1998.tb10494.x

[13] Calmels B Ferguson C Laukkanen MO Adler R Faulhaber M Kim HJ Sellers S Hematti P Schmidt M von Kalle C Akagi K Donahue RE Dunbar CE 2005 Recurrent retroviral vector integration at the Mds1/Evi1 locus in nonhuman primate hematopoietic cells Blood 106 7 2530 2533 15933056 10.1182/blood-2005-03-1115 PMC1895268

[14] Carey BW Markoulaki S Hanna J Saha K Gao Q Mitalipova M Jaenisch R 2009 Reprogramming of murine and human somatic cells using a single polycistronic vector Proceedings of the National Academy of Sciences of the United States of America 106 1 157 162 19109433 10.1073/pnas.0811426106 PMC2629226

[15] Case SS Price MA Jordan CT Yu XJ Wang L Bauer G Haas DL Xu D Stripecke R Naldini L Kohn DB Crooks GM 1999 Stable transduction of quiescent CD34(+)CD38(−) human hematopoietic cells by HIV-1-based lentiviral vectors Proceedings of the National Academy of Sciences of the United States of America 96 6 2988 2993 10077624 10.1073/pnas.96.6.2988 PMC15882

[16] Chang JC Liu D Kan YW 1992 A 36-base-pair core sequence of locus control region enhances retrovirally transferred human beta-globin gene expression Proceedings of the National Academy of Sciences of the United States of America 89 7 3107 3110 1557419 10.1073/pnas.89.7.3107 PMC48813

[17] Chin JY Kuan JY Lonkar PS Krause DS Seidman MM Peterson KR Nielsen PE Kole R Glazer PM 2008 Correction of a splice-site mutation in the beta-globin gene stimulated by triplex-forming peptide nucleic acids Proceedings of the National Academy of Sciences of the United States of America 105 36 13514 13519 18757759 10.1073/pnas.0711793105 PMC2533221

[18] Chui DH Hardison R Riemer C Miller W Carver MF Molchanova TP Efremov GD Huisman TH 1998 An electronic database of human hemoglobin variants on the World Wide Web Blood 91 8 2643 2644 9531571

[19] Ciavatta DJ Ryan TM Farmer SC Townes TM 1995 Mouse model of human beta zero thalassemia: targeted deletion of the mouse beta maj- and beta min-globin genes in embryonic stem cells Proceedings of the National Academy of Sciences of the United States of America 92 20 9259 9263 7568113 10.1073/pnas.92.20.9259 PMC40964

[20] Coffin JM Hughes SH Varmus HE 1997 Retrovirus Cold Spring Harbor Laboratory Press 21433340

[21] Cone RD Weber-Benarous A Baorto D Mulligan RC 1987 Regulated expression of a complete human beta-globin gene encoded by a transmissible retrovirus vector Molecular and cellular biology 7 2 887 897 3029570 10.1128/mcb.7.2.887-897.1987 PMC365147

[22] Crone TM Pegg AE 1993 A single amino acid change in human O6-alkylguanine-DNA alkyltransferase decreasing sensitivity to inactivation by O6-benzylguanine Cancer Res 53 20 4750 4753 8402653

[23] Cunningham MJ Macklin EA Neufeld EJ Cohen AR 2004 Complications of beta-thalassemia major in North America Blood 104 1 34 39 14988152 10.1182/blood-2003-09-3167

[24] Dave UP Akagi K Tripathi R Cleveland SM Thompson MA Yi M Stephens R Downing JR Jenkins NA Copeland NG 2009 Murine leukemias with retroviral insertions at Lmo2 are predictive of the leukemias induced in SCID-X1 patients following retroviral gene therapy PLoS Genet 5 5 e1000491 19461887 10.1371/journal.pgen.1000491 PMC2679194

[25] Dominski Z Kole R 1993 Restoration of correct splicing in thalassemic pre-mRNA by antisense oligonucleotides Proc Natl Acad Sci U S A 90 18 8673 8677 8378346 10.1073/pnas.90.18.8673 PMC47420

[26] Dzierzak EA Papayannopoulou T Mulligan RC 1988 Lineage-specific expression of a human beta-globin gene in murine bone marrow transplant recipients reconstituted with retrovirus-transduced stem cells Nature 331 6151 35 41 2893284 10.1038/331035a0

[27] Emery DW Morrish F Li Q Stamatoyannopoulos G 1999 Analysis of gamma-globin expression cassettes in retrovirus vectors Hum Gene Ther 10 6 877 888 10223722 10.1089/10430349950018283

[28] Emery DW Yannaki E Tubb J Nishino T Li Q Stamatoyannopoulos G 2002 Development of virus vectors for gene therapy of beta chain hemoglobinopathies: flanking with a chromatin insulator reduces gamma-globin gene silencing in vivo Blood 100 6 2012 2019 12200360 10.1182/blood-2002-01-0219

[29] Emery DW Yannaki E Tubb J Stamatoyannopoulos G 2000 A chromatin insulator protects retrovirus vectors from chromosomal position effects Proceedings of the National Academy of Sciences of the United States of America 97 16 9150 9155 10908661 10.1073/pnas.160159597 PMC16837

[30] Evans-Galea MV Wielgosz MM Hanawa H Srivastava DK Nienhuis AW 2007 Suppression of clonal dominance in cultured human lymphoid cells by addition of the cHS4 insulator to a lentiviral vector Mol Ther 15 4 801 809 17299406 10.1038/sj.mt.6300103

[31] Fragkos M Anagnou NP Tubb J Emery DW 2005 Use of the hereditary persistence of fetal hemoglobin 2 enhancer to increase the expression of oncoretrovirus vectors for human gamma-globin Gene therapy 12 21 1591 1600 15944728 10.1038/sj.gt.3302566

[32] Fu XH Liu DP Liang CC 2002 Chromatin structure and transcriptional regulation of the beta-globin locus Experimental cell research 278 1 1 11 12126952 10.1006/excr.2002.5555

[33] Fucharoen S Winichagoon P 2000 Clinical and hematologic aspects of hemoglobin E beta-thalassemia Curr Opin Hematol 7 2 106 112 10698297 10.1097/00062752-200003000-00006

[34] Gardenghi S Marongiu MF Ramos P Guy E Breda L Chadburn A Liu Y Amariglio N Rechavi G Rachmilewitz EA Breuer W Cabantchik ZI Wrighting DM Andrews NC de Sousa M Giardina PJ Grady RW Rivella S 2007 Ineffective erythropoiesis in {beta}-thalassemia is characterized by increased iron absorption mediated by down-regulation of hepcidin and up-regulation of ferroportin Blood 109 11 5027 5035 17299088 10.1182/blood-2006-09-048868 PMC1885515

[35] Gerson SL 2000 Drug resistance gene transfer: Stem cell protection and therapeutic efficacy Exp Hematol 28 12 1315 1324 11146153 10.1016/s0301-472x(00)00548-8

[36] Giardina PJ Grady RW 2001 Chelation therapy in beta-thalassemia: an optimistic update Semin Hematol 38 4 360 366 11605171 10.1016/s0037-1963(01)90030-7

[37] Giardine B van Baal S Kaimakis P Riemer C Miller W Samara M Kollia P Anagnou NP Chui DH Wajcman H Hardison RC Patrinos GP 2007 HbVar database of human hemoglobin variants and thalassemia mutations: 2007 update Hum Mutat 28 2 206 17221864 10.1002/humu.9479

[38] Giardini C Lucarelli G 1994 Bone marrow transplantation in the treatment of thalassemia Curr Opin Hematol 1 2 170 176 9371277

[39] Gorman L Suter D Emerick V Schumperli D Kole R 1998 Stable alteration of pre-mRNA splicing patterns by modified U7 small nuclear RNAs Proc Natl Acad Sci U S A 95 9 4929 4934 9560205 10.1073/pnas.95.9.4929 PMC20190

[40] Greaves DR Fraser P Vidal MA Hedges MJ Ropers D Luzzatto L Grosveld F 1990 A transgenic mouse model of sickle cell disorder [see comments] Nature 343 6254 183 185 2296310 10.1038/343183a0

[41] Grosveld F van Assendelft GB Greaves DR Kollias G 1987 Position-independent, high-level expression of the human beta-globin gene in transgenic mice Cell 51 6 975 985 3690667 10.1016/0092-8674(87)90584-8

[42] Hacein-Bey-Abina S Von Kalle C Schmidt M McCormack MP Wulffraat N Leboulch P Lim A Osborne CS Pawliuk R Morillon E Sorensen R Forster A Fraser P Cohen JI de Saint Basile G Alexander I Wintergerst U Frebourg T Aurias A Stoppa-Lyonnet D Romana S Radford-Weiss I Gross F Valensi F Delabesse E Macintyre E Sigaux F Soulier J Leiva LE Wissler M Prinz C Rabbitts TH Le Deist F Fischer A Cavazzana-Calvo M 2003 LMO2-associated clonal T cell proliferation in two patients after gene therapy for SCID-X1 Science 302 5644 415 419 14564000 10.1126/science.1088547

[43] Han XD Lin C Chang J Sadelain M Kan YW 2007 Fetal gene therapy of alpha-thalassemia in a mouse model Proceedings of the National Academy of Sciences of the United States of America 104 21 9007 9011 17496141 10.1073/pnas.0702457104 PMC1885618

[44] Hanawa H Yamamoto M Zhao H Shimada T Persons DA 2009 Optimized lentiviral vector design improves titer and transgene expression of vectors containing the chicken beta-globin locus HS4 insulator element Mol Ther 17 4 667 674 19223867 10.1038/mt.2009.1 PMC2835111

[45] Hanna J Wernig M Markoulaki S Sun CW Meissner A Cassady JP Beard C Brambrink T Wu LC Townes TM Jaenisch R 2007 Treatment of sickle cell anemia mouse model with iPS cells generated from autologous skin Science 318 5858 1920 1923 18063756 10.1126/science.1152092

[46] Hargrove PW Kepes S Hanawa H Obenauer JC Pei D Cheng C Gray JT Neale G Persons DA 2008 Globin lentiviral vector insertions can perturb the expression of endogenous genes in beta-thalassemic hematopoietic cells Mol Ther 16 3 525 533 18195719 10.1038/sj.mt.6300394

[47] Hongeng S Pakakasama S Chuansumrit A Sirachainan N Sura T Ungkanont A Chuncharunee S Jootar S Issaragisil S 2007 Reduced intensity stem cell transplantation for treatment of class 3 Lucarelli severe thalassemia patients Am J Hematol 82 12 1095 1098 17674372 10.1002/ajh.21002

[48] Imren S Fabry ME Westerman KA Pawliuk R Tang P Rosten PM Nagel RL Leboulch P Eaves CJ Humphries RK 2004 High-level beta-globin expression and preferred intragenic integration after lentiviral transduction of human cord blood stem cells J Clin Invest 114 7 953 962 15467834 10.1172/JCI21838 PMC518665

[49] Imren S Payen E Westerman KA Pawliuk R Fabry ME Eaves CJ Cavilla B Wadsworth LD Beuzard Y Bouhassira EE Russell R London IM Nagel RL Leboulch P Humphries RK 2002 Permanent and panerythroid correction of murine beta thalassemia by multiple lentiviral integration in hematopoietic stem cells Proceedings of the National Academy of Sciences of the United States of America 99 22 14380 14385 12391330 10.1073/pnas.212507099 PMC137892

[50] Kalberer CP Pawliuk R Imren S Bachelot T Takekoshi KJ Fabry M Eaves CJ London IM Humphries RK Leboulch P 2000 Preselection of retrovirally transduced bone marrow avoids subsequent stem cell gene silencing and age-dependent extinction of expression of human beta-globin in engrafted mice PG - 5411-5 Proceedings of the National Academy of Sciences of the United States of America 97 10 5411 5415 10792053 10.1073/pnas.100082597 PMC25842

[51] Karlsson S Bodine DM Perry L Papayannopoulou T Nienhuis AW 1988 Expression of the human beta-globin gene following retroviral-mediated transfer into multipotential hematopoietic progenitors of mice Proceedings of the National Academy of Sciences of the United States of America 85 16 6062 6066 3413076 10.1073/pnas.85.16.6062 PMC281905

[52] Karlsson S Papayannopoulou T Schweiger SG Stamatoyannopoulos G Nienhuis AW 1987 Retroviral-mediated transfer of genomic globin genes leads to regulated production of RNA and protein Proceedings of the National Academy of Sciences of the United States of America 84 8 2411 2415 3470803 10.1073/pnas.84.8.2411 PMC304661

[53] Kim YJ Kim YS Larochelle A Renaud G Wolfsberg TG Adler R Donahue RE Hematti P Hong BK Roayaei J Akagi K Riberdy JM Nienhuis AW Dunbar CE Persons DA 2009 Sustained high-level polyclonal hematopoietic marking and transgene expression 4 years after autologous transplantation of rhesus macaques with SIV lentiviral vector-transduced CD34+ cells Blood 113 22 5434 5443 19339698 10.1182/blood-2008-10-185199 PMC2689045

[54] La Nasa G Argiolu F Giardini C Pession A Fagioli F Caocci G Vacca A De Stefano P Piras E Ledda A Piroddi A Littera R Nesci S Locatelli F 2005 Unrelated bone marrow transplantation for beta-thalassemia patients: The experience of the Italian Bone Marrow Transplant Group Ann N Y Acad Sci 1054 186 195 16339665 10.1196/annals.1345.023

[55] Lacerra G Sierakowska H Carestia C Fucharoen S Summerton J Weller D Kole R 2000 Restoration of hemoglobin A synthesis in erythroid cells from peripheral blood of thalassemic patients Proceedings of the National Academy of Sciences of the United States of America 97 17 9591 9596 10944225 10.1073/pnas.97.17.9591 PMC16909

[56] Leboulch P Huang GM Humphries RK Oh YH Eaves CJ Tuan DY London IM 1994 Mutagenesis of retroviral vectors transducing human beta-globin gene and beta-globin locus control region derivatives results in stable transmission of an active transcriptional structure Embo J 13 13 3065 3076 8039501 10.1002/j.1460-2075.1994.tb06605.x PMC395197

[57] Levasseur DN Ryan TM Pawlik KM Townes TM 2003 Correction of a mouse model of sickle cell disease: lentiviral/antisickling beta-globin gene transduction of unmobilized, purified hematopoietic stem cells Blood 102 13 4312 4319 12933581 10.1182/blood-2003-04-1251

[58] Levasseur DN Ryan TM Reilly MP McCune SL Asakura T Townes TM 2004 A recombinant human hemoglobin with anti-sickling properties greater than fetal hemoglobin J Biol Chem 279 26 27518 27524 15084588 10.1074/jbc.M402578200

[59] Li CL Emery DW 2008 The cHS4 chromatin insulator reduces gammaretroviral vector silencing by epigenetic modifications of integrated provirus Gene therapy 15 1 49 53 17989708 10.1038/sj.gt.3303009

[60] Li CL Xiong D Stamatoyannopoulos G Emery DW 2009 Genomic and functional assays demonstrate reduced gammaretroviral vector genotoxicity associated with use of the cHS4 chromatin insulator Mol Ther 17 4 716 724 19240697 10.1038/mt.2009.7 PMC2835102

[61] Lombardo A Genovese P Beausejour CM Colleoni S Lee YL Kim KA Ando D Urnov FD Galli C Gregory PD Holmes MC Naldini L 2007 Gene editing in human stem cells using zinc finger nucleases and integrase-defective lentiviral vector delivery Nature biotechnology 25 11 1298 1306 10.1038/nbt1353 17965707

[62] Lucarelli G Galimberti M Polchi P Angelucci E Baronciani D Giardini C Politi P Durazzi SM Muretto P Albertini F 1990 Bone marrow transplantation in patients with thalassemia N Engl J Med 322 7 417 421 2300104 10.1056/NEJM199002153220701

[63] Lung HY Meeus IS Weinberg RS Atweh GF 2000 In vivo silencing of the human gamma-globin gene in murine erythroid cells following retroviral transduction Blood Cells Mol Dis 26 6 613 619 11358353 10.1006/bcmd.2000.0343

[64] Luzzatto L 1979 Genetics of red cells and susceptibility to malaria Blood 54 5 961 976 387115

[65] Luzzatto L Goodfellow P 1989 Sickle cell anaemia. A simple disease with no cure [news] Nature 337 6202 17 18 2909889 10.1038/337017a0

[66] Maherali N Sridharan R Xie W Utikal J Eminli S Arnold K Stadtfeld M Yachechko R Tchieu J Jaenisch R Plath K Hochedlinger K 2007 Directly reprogrammed fibroblasts show global epigenetic remodeling and widespread tissue contribution Cell Stem Cell 1 1 55 70 18371336 10.1016/j.stem.2007.05.014

[67] Malik P Arumugam PI Yee JK Puthenveetil G 2005 Successful correction of the human Cooley’s anemia beta-thalassemia major phenotype using a lentiviral vector flanked by the chicken hypersensitive site 4 chromatin insulator Ann N Y Acad Sci 1054 238 249 16339671 10.1196/annals.1345.030

[68] Maquat LE 2005 Nonsense-mediated mRNA decay in mammals J Cell Sci 118 Pt 9 1773 1776 15860725 10.1242/jcs.01701

[69] May C Rivella S Callegari J Heller G Gaensler KM Luzzatto L Sadelain M 2000 Therapeutic haemoglobin synthesis in beta-thalassaemic mice expressing lentivirus-encoded human beta-globin Nature 406 6791 82 86 10894546 10.1038/35017565

[70] May C Rivella S Chadburn A Sadelain M 2002 Successful treatment of murine beta-thalassemia intermedia by transfer of the human beta-globin gene Blood 99 6 1902 1908 11877258 10.1182/blood.v99.6.1902

[71] Miccio A Cesari R Lotti F Rossi C Sanvito F Ponzoni M Routledge SJ Chow CM Antoniou MN Ferrari G 2008 In vivo selection of genetically modified erythroblastic progenitors leads to long-term correction of beta-thalassemia Proceedings of the National Academy of Sciences of the United States of America 105 30 10547 10552 18650378 10.1073/pnas.0711666105 PMC2492493

[72] Miller JL Walsh CE Ney PA Samulski RJ Nienhuis AW 1993 Single-copy transduction and expression of human gamma-globin in K562 erythroleukemia cells using recombinant adeno-associated virus vectors: the effect of mutations in NF-E2 and GATA-1 binding motifs within the hypersensitivity site 2 enhancer [published erratum appears in Blood 1995 Feb 1;85(3):862] Blood 82 6 1900 1906 8400240

[73] Milner PF Clegg JB Weatherall DJ 1971 Haemoglobin-H disease due to a unique haemoglobin variant with an elongated alpha-chain Lancet 1 7702 729 732 4101431 10.1016/s0140-6736(71)91992-1

[74] Murari J Smith LL Wilson JB Schneider RG Huisman TH 1977 Some properties of hemoglobin Gun Hill Hemoglobin 1 3 267 282 19375 10.3109/03630267709003409

[75] Nishino T Tubb J Emery DW 2006 Partial correction of murine beta-thalassemia with a gammaretrovirus vector for human gamma-globin Blood Cells Mol Dis 37 1 1 7 16814578 10.1016/j.bcmd.2006.05.001

[76] Novak U Harris EA Forrester W Groudine M Gelinas R 1990 High-level beta-globin expression after retroviral transfer of locus activation region-containing human beta-globin gene derivatives into murine erythroleukemia cells Proceedings of the National Academy of Sciences of the United States of America 87 9 3386 3390 2333288 10.1073/pnas.87.9.3386 PMC53905

[77] Pabo CO Peisach E Grant RA 2001 Design and selection of novel Cys2His2 zinc finger proteins Annual review of biochemistry 70 313 340 10.1146/annurev.biochem.70.1.313 11395410

[78] Pawliuk R Westerman KA Fabry ME Payen E Tighe R Bouhassira EE Acharya SA Ellis J London IM Eaves CJ Humphries RK Beuzard Y Nagel RL Leboulch P 2001 Correction of sickle cell disease in transgenic mouse models by gene therapy Science 294 5550 2368 2371 11743206 10.1126/science.1065806

[79] Pike-Overzet K van der Burg M Wagemaker G van Dongen JJ Staal FJ 2007 New insights and unresolved issues regarding insertional mutagenesis in X-linked SCID gene therapy Mol Ther 15 11 1910 1916 17726455 10.1038/sj.mt.6300297

[80] Plavec I Papayannopoulou T Maury C Meyer F 1993 A human beta-globin gene fused to the human beta-globin locus control region is expressed at high levels in erythroid cells of mice engrafted with retrovirus-transduced hematopoietic stem cells Blood 81 5 1384 1392 8443396

[81] Porteus MH Carroll D 2005 Gene targeting using zinc finger nucleases Nature biotechnology 23 8 967 973 10.1038/nbt1125 16082368

[82] Puthenveetil G Scholes J Carbonell D Qureshi N Xia P Zeng L Li S Yu Y Hiti AL Yee JK Malik P 2004 Successful correction of the human beta-thalassemia major phenotype using a lentiviral vector Blood 104 12 3445 3453 15292064 10.1182/blood-2004-04-1427

[83] Raftopoulos H Ward M Leboulch P Bank A 1997 Long-term transfer and expression of the human beta-globin gene in a mouse transplant model Blood 90 9 3414 3422 9345024

[84] Ragg S Xu-Welliver M Bailey J D’Souza M Cooper R Chandra S Seshadri R Pegg AE Williams DA 2000 Direct reversal of DNA damage by mutant methyltransferase protein protects mice against dose-intensified chemotherapy and leads to in vivo selection of hematopoietic stem cells Cancer Res 60 18 5187 5195 11016647

[85] Rechavi G Rivella S 2008 Regulation of iron absorption in hemoglobinopathies Curr Mol Med 8 7 646 662 18991651 10.2174/156652408786241401 PMC3722362

[86] Ren S Wong BY Li J Luo XN Wong PM Atweh GF 1996 Production of genetically stable high-titer retroviral vectors that carry a human gamma-globin gene under the control of the alpha-globin locus control region Blood 87 6 2518 2524 8630419

[87] Rivella S Callegari JA May C Tan CW Sadelain M 2000 The cHS4 insulator increases the probability of retroviral expression at random chromosomal integration sites J Virol 74 10 4679 4687 10775605 10.1128/jvi.74.10.4679-4687.2000 PMC111989

[88] Rivella S May C Chadburn A Riviere I Sadelain M 2003 A novel murine model of Cooley anemia and its rescue by lentiviral-mediated human beta -globin gene transfer Blood 101 8 2932 2939 12480689 10.1182/blood-2002-10-3305

[89] Rogers FA Vasquez KM Egholm M Glazer PM 2002 Site-directed recombination via bifunctional PNA-DNA conjugates Proceedings of the National Academy of Sciences of the United States of America 99 26 16695 16700 12461167 10.1073/pnas.262556899 PMC139206

[90] Sabatino DE Seidel NE Aviles-Mendoza GJ Cline AP Anderson SM Gallagher PG Bodine DM 2000a Long-term expression of gamma-globin mRNA in mouse erythrocytes from retrovirus vectors containing the human gamma-globin gene fused to the ankyrin-1 promoter Proceedings of the National Academy of Sciences of the United States of America 97 24 13294 13299 11069298 10.1073/pnas.230453097 PMC27218

[91] Sabatino DE Wong C Cline AP Pyle L Garrett LJ Gallagher PG Bodine DM 2000b A minimal ankyrin promoter linked to a human gamma-globin gene demonstrates erythroid specific copy number dependent expression with minimal position or enhancer dependence in transgenic mice J Biol Chem 275 37 28549 28554 10878017 10.1074/jbc.M004043200

[92] Sadelain M Boulad F Galanello R Giardina P Locatelli F Maggio A Rivella S Riviere I Tisdale J 2007 Therapeutic options for patients with severe beta-thalassemia: the need for globin gene therapy Hum Gene Ther 18 1 1 9 17173507 10.1089/hum.2006.151

[93] Sadelain M Wang CH Antoniou M Grosveld F Mulligan RC 1995 Generation of a high-titer retroviral vector capable of expressing high levels of the human beta-globin gene Proceedings of the National Academy of Sciences of the United States of America 92 15 6728 6732 7624311 10.1073/pnas.92.15.6728 PMC41402

[94] Salvatori F Breveglieri G Zuccato C Finotti A Bianchi N Borgatti M Feriotto G Destro F Canella A Brognara E Lampronti I Breda L Rivella S Gambari R 2009a Production of beta-globin and adult hemoglobin following G418 treatment of erythroid precursor cells from homozygous beta(0)39 thalassemia patients American journal of hematology10.1002/ajh.21539 PMC3572903 19810011

[95] Salvatori F Cantale V Breveglieri G Zuccato C Finotti A Bianchi N Borgatti M Feriotto G Destro F Canella A Breda L Rivella S Gambari R 2009b Development of K562 cell clones expressing beta-globin mRNA carrying the beta039 thalassaemia mutation for the screening of correctors of stop-codon mutations Biotechnology and applied biochemistry 54 1 41 52 19216718 10.1042/BA20080266 PMC3582994

[96] Samakoglu S Lisowski L Budak-Alpdogan T Usachenko Y Acuto S Di Marzo R Maggio A Zhu P Tisdale JF Riviere I Sadelain M 2006 A genetic strategy to treat sickle cell anemia by coregulating globin transgene expression and RNA interference Nature biotechnology 24 1 89 94 10.1038/nbt1176 16378095

[97] Sazani P Kole R 2003 Therapeutic potential of antisense oligonucleotides as modulators of alternative splicing J Clin Invest 112 4 481 486 12925686 10.1172/JCI19547 PMC171400

[98] Schambach A Baum C 2008 Clinical application of lentiviral vectors - concepts and practice Curr Gene Ther 8 6 474 482 19075630 10.2174/156652308786848049

[99] Sierakowska H Sambade MJ Agrawal S Kole R 1996 Repair of thalassemic human beta-globin mRNA in mammalian cells by antisense oligonucleotides Proceedings of the National Academy of Sciences of the United States of America 93 23 12840 12844 8917506 10.1073/pnas.93.23.12840 PMC24007

[100] Silvestroni E Bianco I 1963a [a New Kind of Drepanocytic Anemia: Hemoglobin a-Hemoglobin Lepore Disease.] Progr Med (Napoli) 19 545 548 14122941

[101] Silvestroni E Bianco I 1963b [First Case of the Hb Lepore Disease with Microcythemia Observed in Italy.] Policlinico [Prat] 70 1513 1517 14094667

[102] Sohan K Billington M Pamphilon D Goulden N Kyle P 2002 Normal growth and development following in utero diagnosis and treatment of homozygous alpha-thalassaemia Bjog 109 11 1308 1310 12452474 10.1046/j.1471-0528.2002.01051.x

[103] Steinberg MH Forget BG Higgs DR Nagel RL 2001a Disorders of hemoglobin: Genetics, Pathophysiology and Clinical Management Cambridge, UK Cambridge University Press

[104] Steinberg MH Forget BG Higgs DR Nagel RL 2001b Molecular Mechanism of ß Thalassemia Forget Bernard G Cambridge, UK Cambridge University Press

[105] Suter D Tomasini R Reber U Gorman L Kole R Schumperli D 1999 Double-target antisense U7 snRNAs promote efficient skipping of an aberrant exon in three human beta-thalassemic mutations Hum Mol Genet 8 13 2415 2423 10556289 10.1093/hmg/8.13.2415

[106] Suwanmanee T Sierakowska H Fucharoen S Kole R 2002a Repair of a splicing defect in erythroid cells from patients with beta-thalassemia/HbE disorder Mol Ther 6 6 718 726 12498768 10.1006/mthe.2002.0805

[107] Suwanmanee T Sierakowska H Lacerra G Svasti S Kirby S Walsh CE Fucharoen S Kole R 2002b Restoration of human beta-globin gene expression in murine and human IVS2-654 thalassemic erythroid cells by free uptake of antisense oligonucleotides Mol Pharmacol 62 3 545 553 12181431 10.1124/mol.62.3.545

[108] Svasti S Suwanmanee T Fucharoen S Moulton HM Nelson MH Maeda N Smithies O Kole R 2009 RNA repair restores hemoglobin expression in IVS2-654 thalassemic mice Proc Natl Acad Sci U S A 106 4 1205 1210 19164558 10.1073/pnas.0812436106 PMC2633555

[109] Takahashi K Tanabe K Ohnuki M Narita M Ichisaka T Tomoda K Yamanaka S 2007 Induction of pluripotent stem cells from adult human fibroblasts by defined factors Cell 131 5 861 872 18035408 10.1016/j.cell.2007.11.019

[110] Takahashi K Yamanaka S 2006 Induction of pluripotent stem cells from mouse embryonic and adult fibroblast cultures by defined factors Cell 126 4 663 676 16904174 10.1016/j.cell.2006.07.024

[111] Thomas ED Buckner CD Sanders JE Papayannopoulou T Borgna-Pignatti C De Stefano P Sullivan KM Clift RA Storb R 1982 Marrow transplantation for thalassaemia Lancet 2 8292 227 229 6124668 10.1016/s0140-6736(82)90319-1

[112] Vacek MM Ma H Gemignani F Lacerra G Kafri T Kole R 2003 High-level expression of hemoglobin A in human thalassemic erythroid progenitor cells following lentiviral vector delivery of an antisense snRNA Blood 101 1 104 111 12393543 10.1182/blood-2002-06-1869

[113] Vichinsky EP 2005 Changing patterns of thalassemia worldwide Ann N Y Acad Sci 1054 18 24 16339647 10.1196/annals.1345.003

[114] Weatherall DJ Clegg JB 2001 Inherited haemoglobin disorders: an increasing global health problem Bull World Health Organ 79 8 704 712 11545326 PMC2566499

[115] Wernig M Meissner A Foreman R Brambrink T Ku M Hochedlinger K Bernstein BE Jaenisch R 2007 In vitro reprogramming of fibroblasts into a pluripotent ES-cell-like state Nature 448 7151 318 324 17554336 10.1038/nature05944

[116] Yang B Kirby S Lewis J Detloff PJ Maeda N Smithies O 1995 A mouse model for beta 0-thalassemia Proc Natl Acad Sci U S A 92 25 11608 11612 8524813 10.1073/pnas.92.25.11608 PMC40451

[117] Ye L Chang JC Lin C Sun X Yu J Kan YW 2009 Induced pluripotent stem cells offer new approach to therapy in thalassemia and sickle cell anemia and option in prenatal diagnosis in genetic diseases Proceedings of the National Academy of Sciences of the United States of America 106 24 9826 9830 19482945 10.1073/pnas.0904689106 PMC2701047

[118] Zhao H Pestina TI Nasimuzzaman M Mehta P Hargrove PW Persons DA 2009 Amelioration of murine beta-thalassemia through drug selection of hematopoietic stem cells transduced with a lentiviral vector encoding both gamma-globin and the MGMT drug-resistance gene Blood 113 23 5747 5756 19365082 10.1182/blood-2008-10-186684 PMC2700315

